# Mesenchymal stem cells surpass the capacity of bone marrow aspirate concentrate for periodontal regeneration

**DOI:** 10.1590/1678-7757-2021-0359

**Published:** 2022-04-01

**Authors:** Camila Alves Costa, Tatiana Miranda Deliberador, Rodrigo Paolo Flores Abuna, Thaisângela Lopes Rodrigues, Sérgio Luis Scombatti de Souza, Daniela Bazan Palioto

**Affiliations:** 1 Universidade de São Paulo Faculdade de Odontologia de Ribeirão Preto Departamento de Cirurgia e Traumatologia Buco-Maxilo-Facial e Periodontia Ribeirão Preto São Paulo Brasil Universidade de São Paulo, Faculdade de Odontologia de Ribeirão Preto, Departamento de Cirurgia e Traumatologia Buco-Maxilo-Facial e Periodontia, Ribeirão Preto, São Paulo, Brasil.; 2 Universidade Positivo Faculdade de Ciências da Saúde Departamento de Odontologia Curitiba Paraná Brasil Universidade Positivo, Curitiba, Faculdade de Ciências da Saúde, Departamento de Odontologia, Paraná, Brasil.; 3 Universidade de São Paulo Faculdade de Medicina de Ribeirão Preto Departamento de Bioquímica e Imunologia Ribeirão Preto Brasil Universidade de São Paulo, Faculdade de Medicina de Ribeirão Preto, Departamento de Bioquímica e Imunologia, Projeto Fiocruz-Bi-Institucional de Medicina Translacional, Ribeirão Preto, Brasil.; 4 Universidade Federal de Goiás Faculdade de Farmácia Laboratório de Farmacologia Celular e Toxicologia Goiânia Goiás Brasil Universidade Federal de Goiás, Faculdade de Farmácia, Laboratório de Farmacologia Celular e Toxicologia, Goiânia, Goiás, Brasil.

**Keywords:** Root cementum, Periodontal regeneration, Tissue engineering, Stem cells

## Abstract

Regenerative approaches using mesenchymal stem cells (MSCs) have been evaluated to promote the complete formation of all missing periodontal tissues, e.g., new cementum, bone, and functional periodontal ligaments. MSCs derived from bone marrow have been applied to bone and periodontal defects in several forms, including bone marrow aspirate concentrate (BMAC) and cultured and isolated bone marrow mesenchymal stem cells (BM-MSCs). This study aimed to evaluate the periodontal regeneration capacity of BMAC and cultured BM-MSCs in the wound healing of fenestration defects in rats. Methodology: BM-MSCs were obtained after bone marrow aspiration of the isogenic iliac crests of rats, followed by cultivation and isolation. Autogenous BMAC was collected and centrifuged immediately before surgery. In 36 rats, fenestration defects were created and treated with suspended BM-MSCs, BMAC or left to spontaneously heal (control) (N=6). Their regenerative potential was assessed by microcomputed tomography (µCT) and histomorphometry, as well as their cell phenotype and functionality by the Luminex assay at 15 and 30 postoperative days. Results: BMAC achieved higher bone volume in 30 days than spontaneous healing (p<0.0001) by enhancing osteoblastic lineage commitment maturation, with higher levels of osteopontin (p=0.0013). Defects filled with cultured BM-MSCs achieved higher mature bone formation in early stages than spontaneous healing and BMAC (p=0.0241 and p=0.0143, respectively). Moreover, significantly more cementum-like tissue formation (p<0.0001) was observed with new insertion of fibers in specimens treated with BM-MSCs within 30 days. Conclusion: Both forms of cell transport, BMAC and BM-MSCs, promoted bone formation. However, early bone formation and maturation were achieved when cultured BM-MSCs were used. Likewise, only cultured BM-MSCs were capable of achieving complete periodontal regeneration with inserted fibers in the new cementum-like tissue.

## Introduction

Restoring periodontal structures functionally lost by periodontal disease remains one of the major challenges in Periodontology due to the limited capacity of the periodontium tissues for self-regeneration and the complexity of their architecture and microenvironment.^1^ Regenerative approaches, including the use of bone replacement grafts, guided tissue regeneration (GTR), and various growth factors and/or host modulating agents, such as enamel protein derivatives, have shown some efficacy in achieving only partial reconstruction of bone defects, especially in advanced cases.^1^

New techniques and treatment options involving tissue engineering and cell-based therapies to promote regeneration of periodontal and bone defects have been evaluated.^1^ The literature shows that mesenchymal stem cells (MSCs) can be advantageous for periodontal regeneration and promising for tissue regeneration in clinical applications.^2,3^

MSCs are multipotent cells that can adhere to plastic, differentiate into many tissues, and show a specific surface antigen expression, including CD90+ and CD45-.^4^ These cells can be obtained from different sources in the adult organism,^2^ including bone marrow.^5^ MSCs derived from bone marrow can be used in several ways, such as bone marrow aspirates (BMA),^6^ bone marrow aspirate concentrates (BMAC)^7-9^ or cultured and isolated bone marrow mesenchymal stem cells (BM-MSCs).^9-16^

BM-MSCs used in periodontal defects to achieve regeneration have shown promising but conflicting results.^2^ Clinically, a recent systematic review only showed clinical reports, case series, and phase I/II trials using BM-MSCs in intrabony or furcation defects, with substantial variation in terms of reduction and gain of clinical attachment.^17^ Therefore, most of the literature on the use of BM-MSCs is focused on preclinical studies to gain a greater understanding of the action and effect of their use to further promote their application in clinical practice. Thus, further preclinical research on cell therapy is encouraged.^1^

Preclinical studies have investigated the effects of BM-MSCs on bone, cementum, and periodontal ligament regeneration.^9-16^ The use of BM-MSCs has achieved reliable results in terms of bone regeneration, such as the increase of local bone density in the subperiosteal buccal alveolar bone surface at the tooth extraction site.^18^ Few studies have shown the induction of cementum formation in fenestration defects.^9-11^ However, the implementation of these delivery-isolated BM-MSCs in periodontal therapy is hampered by inbuilt difficulties in the isolation, characterization, and identification of the appropriate *ex vivo* culture before reimplantation.^1^ Moreover, this process remains expensive, difficult, and requires specialized labor and highly sophisticated laboratories.^19^

BMACs are another source of bone marrow-derived mesenchymal stem cells. Although the percentage of MSC content in BMAC represents a very limited fraction of the implanted cells,^19^ its manipulation and implantation are simplified and less expensive than isolated BM-MSCs. To produce BMAC, after bone marrow collection, the tissue only needs to be centrifuged twice: first to remove red cells, and second, to separate the platelets and nucleate cells from the platelet-poor plasma.^7^ Therefore, BMAC represents a minimally manipulated whole tissue fraction, which preserves the physiological microenvironment of multiple cell types, such as platelets that are rich in growth factors in their natural proportions.^7,19^

Promising results have been shown when BMAC is used to regenerate different bone sites.^8,9,20-22^ In oral sites, BMAC, associated with allogeneic bone-combined and recombinant human bone morphogenetic protein-2, have shown 90% and 100% effectiveness (in terms of adequate grafted bone height and width for dental implants), respectively, in the reconstruction of large continuous mandibular defects (N=31) after six months.^23^ BMAC also favors the presence of high amounts of stem and progenitor cells. Among them, endothelial cells play an essential role in angiogenesis.^24^ However, the use of BMAC to treat periodontal defects is still poorly described.

This study aimed to evaluate the performance of BMAC in terms of periodontal regeneration in a fenestration rat model and compare it with the isolated suspension form of BM-MSCs.

## Methodology

### Animals

All animal procedures were performed in accordance with the Committee of Ethics in Animal Research at our institution (Protocol 2014.1.494.58.0). This article was written following ARRIVE guidelines.^25^

In total, 36 old male isogenic Wistar Kyoto rats weighing 250–300 g were used, and all procedures were done under intramuscular anesthesia (ketamine, 7 mg/100 g of body weight; xylazine, 0.6 mg/100 g of body weight). Animals were randomly assigned to one of three experimental groups: i) spontaneous healing – Control, ii) BM-MSCs, and iii) BMAC. Each group was further divided into two randomized subgroups according to two healing time points: 15 and 30 days. The number of animals per group was six (N=6) at each time point, and each animal was an experimental unit. All analyses were performed on animals from all groups (N=6).

### BM-MSCs collection and isolation

**Figure 1:**
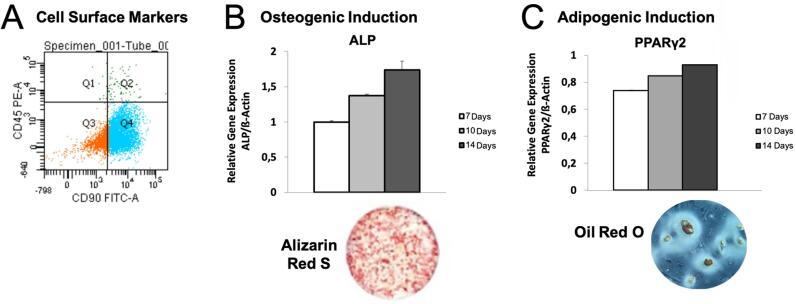
Graphs representing the characterization of bone marrow mesenchymal stem cells (BM-MSCs). (A) Cell surface markers CD45 and CD90 in BM-MSCs. Blue represents the selected BM-MSCs used in the study. (B) Representative wells and relative gene expression of the capacity for osteogenic induction in Alizarin Red S assay and APL gene expression. (C) Representative wells and relative gene expression of the capacity of adipogenic induction in Oil Red O assay and PPARγ2 gene expression

From every iliac crest from two isogenic rats, 0.5 mL of bone marrow was aspirated using a 10-mL sterile syringe, containing 0.1 mL of heparin.^7^ Cells were isolated with Ficoll-Paque PREMIUM (Gibco-Invitrogen, Grand Island, NY) and cultured in flasks with Dulbecco’s Modified Eagle’s Medium (D-MEM) (Gibco-Invitrogen), supplemented with 10% fetal bovine serum (FBS) (Gibco-Invitrogen), 50 μg/mL of gentamicin (Gibco-Invitrogen), and 3 μg/mL of fungizone (Gibco-Invitrogen). Adherent cells at 90% confluence were enzymatically detached, incubated with monoclonal anti-rat antibodies CD45 (Mouse Anti-Rat PE, Santa Cruz Biotechnology, Santa Cruz, CA) and CD90 (FITC Mouse Anti-Rat, Santa Cruz Biotechnology), and sorted by flow cytometry (FACS ARIA III, BD Bioscience, San Jose, CA) to obtain a subpopulation of CD45-CD90+ BM-MSCs, which were kept at 37ºC, with 5% CO_2_ in flasks with growth medium. BM-MSCs showed high unmarked cells for CD45 (73.9%) and marked cells for CD90 (0.5%) (Figure 1A). The medium was changed three times a week, and only cells at passage 8 were used. To show capacity of differentiation, cells at passage 8 were subjected to osteogenic and chondrogenic induction. BM-MCSs were capable of expressing the osteoblast phenotype, corroborated by matrix mineralization deposits and alkaline phosphatase (ALP) expression (Figure 1B). The adipogenic phenotype was confirmed by lipid accumulation and PPARγ2 expression (Figure 1C).

#### Osteogenic induction.

BM-MSCs were seeded in 24-well plates (2 × 10^4^ cells/well) in an osteogenic medium consisting of α-Modified Eagle Medium (α-MEM, Gibco-Invitrogen), supplemented with 10% FBS and 100 units/mL penicillin (Gibco-Invitrogen), 100 μg/mL streptomycin (Gibco-Invitrogen), 3 μg/mL fungizone (Gibco-Invitrogen), 5 μg/mL ascorbic acid (Sigma-Aldrich), 7 mM ß-glycerophosphate (Sigma-Aldrich), and 10^-7^ mM dexamethasone (Sigma-Aldrich). After 21 days, extracellular matrix mineralization was assessed using Alizarin Red (Sigma-Aldrich) staining.^26^ To evaluate the expression of ALP, cells were seeded in 6-wells plates (1 x 10^5^ cells/well) in osteogenic medium, and the total RNA was extracted after seven, 10, and 14 days using SV Total RNA Isolation Systems (Promega Corporation, WI, USA) according to the manufacturer’s instructions. RNA integrity was assessed using a 2100 Bioanalyzer (Agilent Technologies, CA, USA). Only samples with RIN values ≥ 8 were used to generate complementary DNA (cDNA) from 1 μg of RNA using a High-Capacity cDNA Reverse Transcription Kit (Applied Biosystems, CA, USA). Quantitative real-time polymerase chain reaction (RT-PCR) was carried out using TaqMan PCR Master Mix (Applied Biosystems), and relative gene expression of ALP (Rn01516028_m1, Thermo Fisher Scientific, Waltham, Massachusetts, EUA) was normalized to β-actin expression using the comparative threshold method (2^-ΔΔCt^).^27^

#### Adipogenic induction.

BM-MSCs were seeded in 24-wells plate (2 × 10^4^ cells/well) in an adipogenic medium containing a growth medium supplemented with 10^-6^ M dexamethasone, 0.5 mM 3-isobutyl-1-methylxanthine (Sigma-Aldrich), 10 mg/mL insulin (Sigma-Aldrich), and 0.1 M indomethacin (Sigma-Aldrich). After 10 days, lipid accumulation was evaluated using Oil Red O (Sigma-Aldrich) staining.^28^ To evaluate the expression of peroxisome proliferator-activated receptor gamma-2 (PPAR_γ_2, Rn00440945_m1, Thermo Fisher Scientific), cells were seeded in 6-well plates (1 × 10^5^ cells/well) in an adipogenic medium, and the total RNA was collected after seven, 10, and 14 days to carry out the RT-PCR, as described above.

### BMAC collection

BMACs were collected and prepared for each rat at the time of surgery. After anesthesia, 0.5 mL of bone marrow was aspirated from the iliac crest of each rat using a 10-mL sterile syringe containing 0.1 mL of heparin (to prevent clotting) and stored in sterile microtubes. The concentration of the bone marrow aspirate (BMA) was determined using a previously described protocol.^7^ Briefly, microtubes were centrifuged at 160 × *g* for 20 min at 22°C to separate the plasma, which contained platelets and mononuclear cells (upper layer), from the red cells. This upper layer, containing mononuclear cells and platelets, was centrifuged again at 400 × *g* for 15 min at 22°C. The supernatant containing the platelet-poor plasma was removed, leaving the BMAC, which contained the platelet-rich plasma remaining in the bone marrow and the buffy coat, rich in bone marrow mononuclear cells. Immediately before application into the defect, quantification of nucleated bone marrow cells and platelets was manually performed in a Neubauer chamber using Trypan Blue or after dilution and erythrocyte lysis with Brecher liquid, respectively. The average platelet and nucleated cells counted in the BMAC samples were 296.40±69.25 × 10^3^ platelets/μL and 100.87±27.76 × 10^3^ nucleate cells/μL, respectively. Only samples containing viable cells were used in this study.

### *In vivo* experimental surgery

All surgical procedures were performed under a 10–40× magnifying stereomicroscope (Nikon SMZ800, Nikon Instruments Inc., Tokyo, Japan) to identify anatomic landmarks. The same trained operator performed the procedures to reduce risk of bias. Both sides of the mandible underwent the same treatment.

After intramuscular anesthesia, surgical sites were shaved off and disinfected. A superficial extraoral incision was made at the base of the mandible to expose the bones. Periodontal fenestration defects of 2 mm, 4 mm, and 1 mm in height, length, and depth, respectively, were created to denude the distal and buccal (mesial length limit) roots of the first molar and the mesial root of the second molar (distal length limit) using a round bur with high-speed instrumentation under irrigation.^29^ The height limit of the defect was 1 mm above and below the center of the defect.

Defects were filled with one of the treatments or left to spontaneously heal (control). Isogenic BM-MSCs (derived from the pooled BM-MSCs from the two rats excluded from surgical procedures) were applied at 1.2 × 10^6^ cells in 25 µL of pellet-suspension with phosphate-buffered saline (PBS, Sigma-Aldrich). The cell density chosen was based on previous studies that showed the regeneration capacity of BM-MSCs.^13-16^ BMAC was obtained immediately after centrifugation of the bone marrow aspirate from each animal; an autogenous application, as described above. The quantity of the applied BMAC varied from each specimen to obtain the same concentration of 1.2 × 10^6^ BM-MSCs, varying from 7.54 µL to 16.66 µL, and was completed with PBS to a final volume of 25 µL. A 25-µL pellet-suspension of BM-MSCs and BMAC was used to provide full filling of the defect, based on a previous study that used a similar periodontal model.^7^ Soft tissues (muscle and skin) were sutured in layers, and an intramuscular injection of 24.000 unities of Penicillin G-benzathine (Zoetis, NJ, USA) and 0.08 mL of analgesic (Lema-injex Biologic, SP, BR) 0.5 g/mL were applied.

At the end of each experimental period, the animals were anesthetized and euthanized in a 100% CO_2_-filled chamber. All mandibles were fixed in 4% formaldehyde for 24 h for micro-computerized tomography (µCT) or histological and histomorphometric analyses. In the left mandible, before fixation to µCT, soft tissues near the surgical area were carefully collected, preserving the adjacent periosteum, immediately stocked in cold PBS with 2 µL of protease inhibitor (1:200; complete™, Roche, Basel, Switzerland), and processed as previously described for the Luminex assay.^30^

### µCT analysis

Non-demineralized mandibles were scanned using a cone-bean µCT Skyscan 1174 scanner (Bruker, Billerica, MA, USA). The X-ray generator was operated at an accelerated potential of 60 kV with a beam current of 165 μA and exposure time of 650 ms per projection at 180°. Images were recorded with a voxel size of 6 μm × 6 μm × 6 μm. The tomographic projections were rebuilt (N. Recon, Version 1.66, Bruker) to generate 3D images. A volume of interest (VOI) was outlined from analyses of scanned images in 2D on the coronal, transaxial, and sagittal axes (DataViewer, Bruker) regarding the following sequence: region of interest, which was chosen individually following the limit of the distal margin of the mesial root from the first molar to the distal margin of the mesial root of the second molar, with the beginning of the dental roots present in the defect as the depth limit, followed by interpolation and binarization of the images. For each sample, a volumetric software (3D analyses, CTAn v.1.10) was used to record the Percent Bone Volume (Bv/Tv), which is the percentage (%) of new bone volume (mm^3^) of the total new tissue volume of the defect (mm^3^). Therefore, for each specimen, Bv/Tv represented the percentage of new bone formation in the entire defect. The non-bone tissue in the defect represents the newly formed connective tissue, characterized by µCT as a radiolucent area, e.g., empty.

### Descriptive histological and histomorphometry analyses

Following fixation, right mandibles were decalcified in a 10% ethylenediaminetetraacetic acid solution ( Merck Millipore, Darmstadt, Germany). After this procedure, transverse serial sections of 5 μm thickness were obtained in a corono-apical sequence, and blades were stained with hematoxylin-eosin (HE, Merck Millipore).

From the middle of the defect, six histological sections were captured using a light microscope (Leica DM LB2 Microsystems, Wetzlar, GmbH, Germany; Leica DC 300F Microsystems). The formation of new connective tissue, bone, and cementum, the presence and orientation of the fibers of the periodontal ligament (PDL), and the presence or absence of dentoalveolar ankylosis, inflammatory reaction, and radicular resorption were evaluated. Cementum extension (CE), which is the linear extension of the new cementum in the total extension of the distal root of the first molar, was performed at 40× magnification in the image software ImageJ^®^ 1.6.0 (Wayne Rasband (NIH), Bethesda, MA, USA).

### Luminex assay

In soft tissue biopsies, bone and cementum regeneration-related molecules, namely sclerostin, osteoprotegerin (OPG), osteopontin (OPN), and osteocalcin (OCN), were quantified using the Luminex xMAP assay via a Milliplex^®^ kit (RBN2MAG-31K-04 Rat Bone Magnetic Panel 2) following the manufacturer’s recommendations. The level of each molecule, expressed as pg/mg, was normalized by the amount of total protein in each sample using a commercial kit (BioRad, Hercules, CA, USA), according to the manufacturer’s instructions. The results are shown in pg/mL.

### Statistical analyses

Animal sample size was determined using the G*Power software (Heinrich Heine Universität Düsseldorf), considering previous studies with similar regeneration periodontal defects and bone volume as their primary outcomes.^31-34^ A sample size of six animals was used, and each animal was the experimental unit, allocated to each group according to the number of caged animals.

Data were analyzed using the Prism 7 software (GraphPad, La Jolla, CA, USA). Differences among the groups were evaluated with a t-test or two-way analysis of variance (ANOVA) and Tukey’s post-hoc test. Statistical significance was set at p<0.05.

## Results

### Bone volume

**Figure 2:**
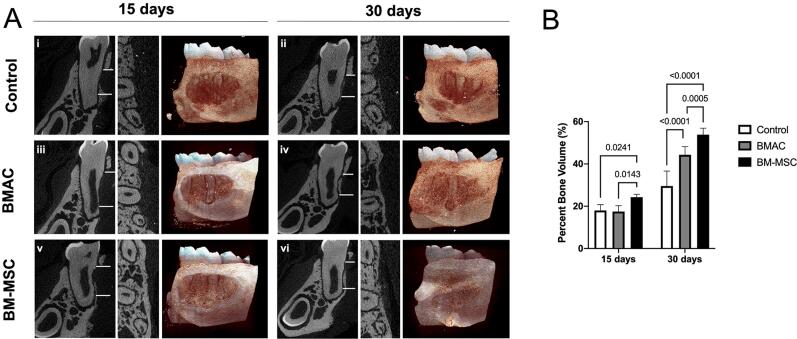
Micro-computerized tomography (μCT) results of the Control, BMAC, and BM-MSC groups in the regeneration of fenestration periodontal defects in 15 and 30 days after surgery. (A) μCT images in 2D longitudinal and coronal axis and 3D reconstructed images within 15 and 30 days of spontaneous healing (i, ii), and BMAC (iii, iv) and BM-MSCs (v, vi) treatments. White lines represent the longitudinal limits of the defect. (B) Histogram reporting the bone volume percentage (Bv/Tv, %). Considering α=5%, the presence of BM-MSCs in suspension form significantly affected bone formation in 15 and 30 days. BMAC also influenced bone volume within 30 days compared to spontaneous healing. Means and standard deviations of the groups and experimental times: 15 days: Control (18.01±2.71), BMAC (17.52±2.74), BM-MSC (24.27±1.38); 30 days: Control (29.55±7.08), BMAC (44.32±3.83), BM-MSC (53.87±3.04)

According to the µCT analysis, the percentage of bone volume increased in all groups at 15 and 30 days (p<0.001). Defects filled with BM-MSCs showed higher bone formation at 15 (Bv/Tv_BM-MSCs_=24.27 mm^3^±1.38) and 30 days (Bv/Tv_s_BM-MSCs=53.87 mm^3^±3.04) than the Control (Bv/Tv_Control_=18.01 mm^3^±2.71, p=0.0241 in 15 days; Bv/Tv_Control_=29.55 mm^3^±7.08, p<0.0001 in 30 days) and BMAC (Bv/Tv_BMAC_=17.52 mm^3^±2.74, p=0.0143 in 15 days; Bv/Tv_BMAC_=44.32 mm^3^±3.83, p=0.0005 in 30 days) (Figure 2). Defects filled with BMAC showed statistically higher bone formation at 30 days (p<0.0001) than spontaneous healing (control) (Figure 2).

### Defect closure, new cementum, and periodontal regeneration

All slides were assessed by a single blinded examiner. Healing occurred uneventfully, and no intense inflammatory reaction, ankylosis, or root resorption was observed during the entire period.

**Figure 3:**
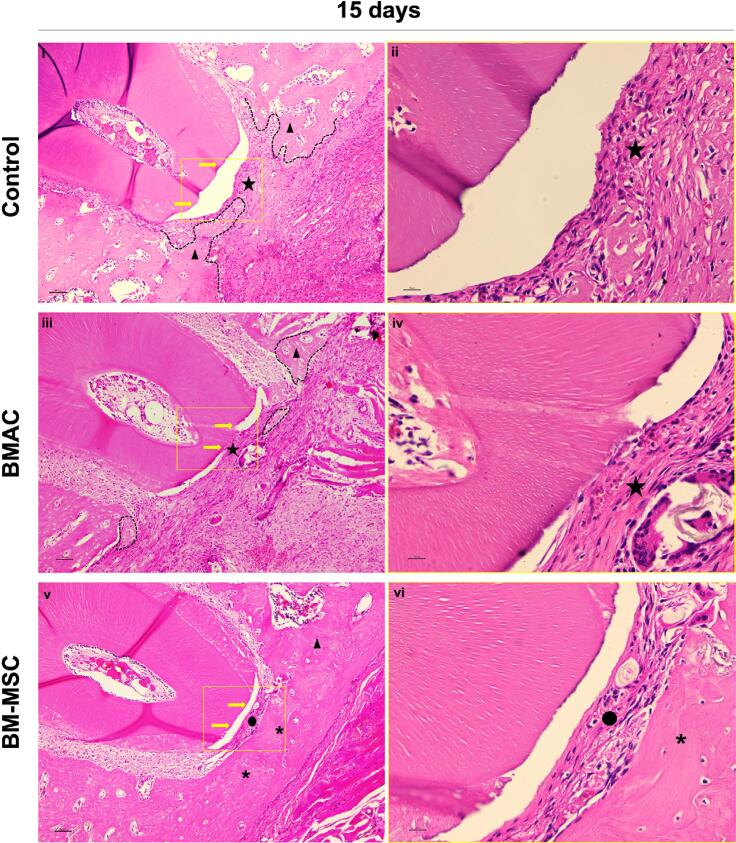
Approximated histological view of the center of the periodontal fenestration defect within 15 days of the spontaneous healing (i, ii), and BMAC (iii, iv) and BM-MSCs (v, vi) treatments (H.E., original magnification 10× and 40×). Yellow rectangles represent the area of magnification. Yellow arrows represent the denuded distal root of the first molar in the center of the defect. Dashed lines delineate the new bone formed into the defect from the edges to the center of the no-closed defects. Stars represent rich connective tissue in the center of the no-closed defects in the Control and BMAC groups. Triangles indicate new immature bone and asterisks indicate new regular mature bone. Circles represent new periodontal ligaments with non-inserted new fibers disposed in parallel in the BM-MSCs group. White arrows indicate the thin layer of new cementum-like tissue

*15 days* (Figure 3) — It was observed that in most specimens, all groups showed new immature, irregular, and thick bone formation partially filling the periodontal defect from the edges to the center. In these specimens, connective tissue was visualized in the center of the defect under the distal root of the first molar. In some specimens treated with BM-MSCs, defects were closed with new bone and a new periodontal ligament containing parallel fibers under the dental root. In this group, some areas of mature bone with a regular and dense bone matrix and osteocytes were observed. No new cementum was observed in any group.

**Figure 4:**
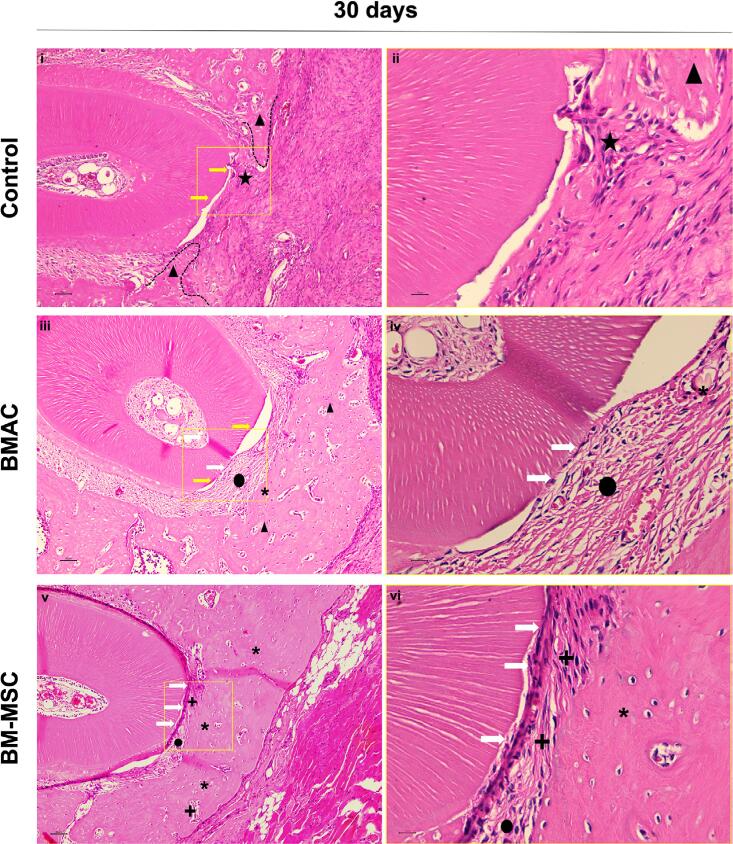
Histological view of the center of the periodontal fenestration defect within postoperative 30 days of spontaneous healing (i, ii),and BMAC (iii, iv) and BM-MSCs (v, vi) treatments (H.E., original magnification 10× and 40×). Yellow rectangles represent the area of magnification. Yellow arrows represent the denuded distal root of the first molar in the center of the defect. Dashed lines delineate the new bone formed into the defect from the edges to the center of the no-closed defects in the Control group. Stars represent rich connective tissue in the center of the no-closed defects in the Control group. Triangles indicate new immature bone and asterisks indicate new regular mature bone. Circles represent new periodontal ligament with non-inserted new fibers disposed of at parallel. A complete periodontal regeneration is shown in the BM-MSCs group (v, vi). White arrows indicate the thin layer of new cellular cementum-like tissue. A plus sign represents the new periodontal ligament with inserted fibers, obliquely disposed in the new cementum

*30 days* (Figure 4) — Specimens in the control group showed only partial bone neoformation without areas with mature bone and remaining connective tissue in the center of the defect. All defects filled with BMAC or BM-MSCs showed new bone filling almost in the entire defect and a new periodontal ligament under the dental root. Areas with regular and mature bone were observed in the specimens treated with BMAC and, more frequently, with BM-MSCs. Despite having been closed with new bone, defects filled with BMAC presented no significant new cementum formation, showing a new periodontal ligament with collagen fibers arranged in parallel to the root surface in most specimens. Only one specimen from the BMAC group had a thin layer of new cement partially covering its dental root (36% of the root with cementum in one specimen). All specimens filled with the BM-MSCs showed new cementum formation in some extension of the distal root of the first molar, and two specimens had 100% of the root covered with new cementum. This new cementum was defined as a cellular amorphous matrix deposited on the root surface with a distinct reverse line separating the new cement from the dentin below, with fibers obliquely inserted in the root.^7^ Then, only in specimens in the BM-MSC group complete periodontal regeneration was achieved, represented by fibers of the new periodontal ligament arranged in parallel and obliquely/perpendicularly to the thin layer of new cementum.

**Figure 5:**
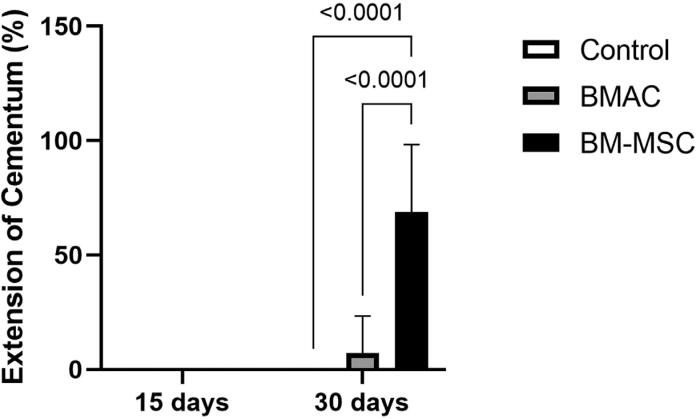
Histogram reporting the histomorphometry analysis of cementum extension (%) - a linear extension of the new cementum in the total extension of the distal root of the first molar - at postoperative 15 and 30 days. Filling with BM-MSCs affects cementum formation within 30 days. Means and standard deviations of the groups at different experimental times: 15 days: no cementum-like tissue was observed in any group; 30 days: Control (zero), BMAC (7.2±16.10), BM-MSCs (68.83±29.53)

*Cementum extension-CE* (Figure 5): BM-MSCs showed statistical significance in the EC compared with other groups at 30 days (EC_Control_=0, EC_BMAC_=7.2%±16.10, EC_BM-MSCs_=68.83±29.53, p<0.0001).

### Mature bone and cementum phenotype

**Figure 6:**
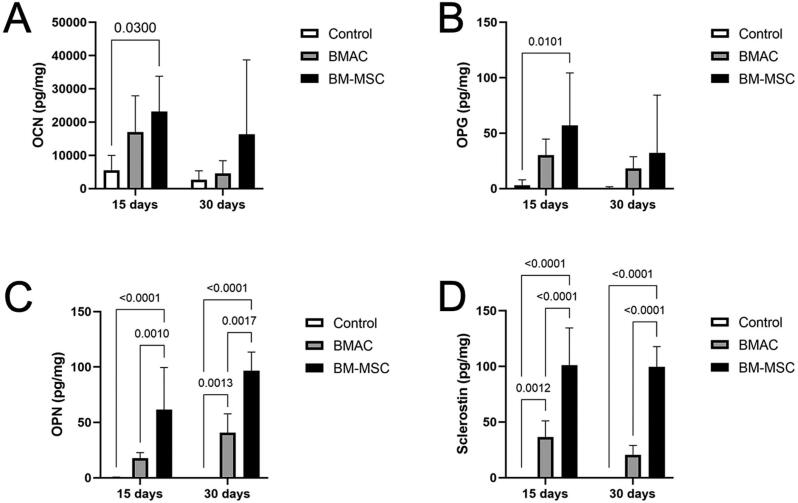
Cell phenotype investigation in Luminex assay within 15 and 30 days of spontaneous healing, and BMAC and BM-MSCs treatments. Histograms reporting (A) osteocalcin (OCN), (B) osteoprotegerin (OPG), (C) osteopontin (OPN), and (D) sclerostin levels

To evaluate the phenotype of the cells involved in graft consolidation, a Luminex assay was performed (Figure 6).

Defects filled with BM-MSCs showed a higher level of OCN after 15 days than the spontaneous healing group (p=0.03), consisting of more osteoblasts with mature bone-deposing capacity in the early regeneration time. Likewise, higher levels of OPG showed earlier commitment of the osteoblastic population in the BM-MSC group than in the control group (p=0.0101). Moreover, a phenotype close to maturity was found earlier in defects filled with BM-MSCs. This was seen by the higher OPN level in the BM-MSC group than in the control and BMAC groups after 15 days (p<0.0001 and p=0.0010, respectively), maintaining the results at 30 days (p<0.0001 and p=0.0017, respectively). Despite not achieving a statistically significant difference, defects filled with BMAC also showed higher levels of OCN, OPG, and OPN than the control group, especially 15 days after surgery, which supports bone formation results. Moreover, after 30 days, more mature bone could be found in defects filled with BMAC than in those which spontaneously healed, which can be attributed to the higher level of OPN at this time point (p=0.0013). Few detections of OPN in the control group support the absence of mature bone found in the histological analysis. Ultimately, superior regulation of bone remodeling and formation of reparative cementum was also shown in the defects filled with BM-MSCs at 15 and 30 days after surgery, represented by the highest level of sclerostin (p<0.0001). The BMAC group also showed a higher level of sclerostin than the control group at 15 days (p=0.0012).

## Discussion

This study investigated the periodontal tissue regenerative capacity of cultured BM-MSCs, compared with BMAC, in a rat tissue fenestration model. Data showed that BMAC induces bone, but not cementum formation. In contrast, BM-MSCs cause faster bone formation and maturation, and successful periodontal regeneration, including the cementum and inserted fibers of the new periodontal ligament. Moreover, earlier features of differentiation were observed with the use of cultured BM-MSCs but not with aspirated forms of bone marrow.

The purpose of using BMAC is to obtain MSCs which are less expensive and laborious, and show greater clinical application. The promising bone formation attribute of BMAC, mainly due to the concentration of mononuclear cells that can enhance differentiation into osteoblasts and, consequently, improve osteogenesis,^20,24^ has been shown in previous studies,^8,19,20,22,24^ In our study, BMAC bone regeneration capacity could be observed, although it was unable to induce cementum formation. This concentrated form of bone marrow aspirate could reach bone maturation in 30 days, compared to spontaneous healing. Besides representing an important result, considering the regeneration of bone defects in which direct impact could be observed, some aspects need to be considered when using BMAC in clinical settings, including the difficulty of controlling cell density and viability for clinical implantation,^19^ the need for a second surgical site, and patient morbidity.

None of the specimens showed cementum formation 15 days after surgery, and when BMAC was applied, only one specimen showed 36% of the root with cementum formation 30 postoperative days. A previous study using BMAC showed a thin layer of a cementoid-like matrix on the root surface only 30 days after application.^7^ However, in contrast with our findings, the authors showed new cementum in all the specimens, and no statistical differences were found in the amount of new bone formation, OCN-positive cells, and TRAP-positive cells between spontaneous healing and BMAC groups.

We can speculate that these distinctive results, in terms of periodontal regeneration, occur because of the direct impact of the extra BMAC components on the differentiation process. To obtain the concentration of MSCs in BMA, other nucleated cells, platelets, cytokines, and growth factors are also retained.^35^ Theoretically, this could favor the regeneration process. However, these extra components can inhibit, downregulate or centralize cell differentiation in fewer cell types. The centrifugation process used in this study increased the number of nucleated bone marrow cells by four times. Moreover, although statistically insignificant, higher levels of OPN, OCN, and OPG were observed in the BMAC group than in the spontaneous healing group. Therefore, this concentration of nucleated cells, in the presence of molecules related to bone formation, could explain the tendency for bone formation when BMAC was applied in our study.

 The application of BM-MSCs in suspension results in faster bone regeneration in fenestration defects. It was expected that the transplantation of these multipotent MSCs may induce early differentiation signals in the defect microenvironment and play a critical role in cellular interaction with host MSCs to generate periodontal tissues.^10^ The application of BM-MSCs seemed to induce bone formation with a phenotype close to maturity at earlier time points in this study. This was shown by the higher levels of molecules related to the deposition of mature bone osteoblasts, such as OCN, OPN, and OPG, especially in early periods - 15 postoperative days - in the BM-MSC group.

Importantly, the use of BM-MSCs in suspension form in this study showed better results in terms of new cementum formation, and higher levels of sclerostin were also found, compared to the control and BMAC groups. Sclerostin, the product of the *SOST* gene, is a glycoprotein that plays an important role in controlling bone remodeling as an osteocyte-derived signal.^36^ Its expression has also been found in the lacunae of cementocytes.^36^ Moreover, OCN, OPN, and sclerostin are secreted by cementoblasts and have been critical in the formation of reparative cementum.^1,36^

These results are consistent with our histologic and histomorphometric findings. A thin layer of new cellular cementum and a new PDL with obliquely oriented fibers were observed in defects filled with isolated BM-MSCs. Nonetheless, the use of BM-MSCs in suspension resulted in considerable earlier formation of mature bone and was able to lead to true periodontal regeneration on postoperative day 30. These findings are corroborated by other studies.^10-13,15,16^ Paknejad, et al.^15^ (2015) found that 80% of the height of three-wall intrabony defects was filled by new cementum after the transplantation of cultured BM-MSCs in an organic bovine bone mineral in a canine model with induced periodontitis.

Some conflicting results can be found in the literature regarding the use of BM-MSCs for periodontal regeneration, hampered by inherent difficulties in the stage of differentiation, lineage, and heterogeneity, as well as the number of cells transferred to the defects.^2^ We can consider that the protocol used in this study was efficient. Isolated and cultured BM-MSCs, when applied to fenestration defects at a density of 1.2 x 10^6^ cells, could promote complete periodontal regeneration. This cell density was selected based on previous studies that showed the regeneration capacity of BM-MSCs.^13-16^ These studies used a larger number of cells than those used in this study (varying from 2 × 10^6^ to 2 × 10^7^). However, reduced cell survival and, consequently, reduced regeneration capacity can occur when high concentrations of MSCs are used due to greater difficulty in inserting nutrients into the deepest areas of the periodontal defects.^37^ Therefore, after a pilot study, 1.2 x 10^6^ cells were considered sufficient to achieve periodontal regeneration without hindering cell capacities in this type of defect. Many studies have used carriers and biomaterial scaffolds to hold and support MSCs. However, these carriers can help or interfere with new tissue formation, favoring the abundant presence of multinucleated giant cells.^14^ Therefore, because this pre-clinical study is a proof of principle, BM-MSCs were applied alone, in suspended form, to avoid any influence of carriers on the periodontal tissue formation capacity and, therefore, facilitate the real influence of BM-MSCs on the morphological and molecular aspects of periodontal regeneration. While promising results were found in the use of BM-MSCs, including their potential to communicate with dental tissue and lead to periodontal regeneration, future translational studies using other vehicles can verify the best way to support these cells in large periodontal defects.

To the best of our knowledge, this is the first study in the literature that aims to comparatively test the *in vivo* effects of BMAC and BM-MSCs. Since periodontal fenestration defects are not critical, our results indicate a key role of MSC derivatives from the bone marrow in providing support for faster periodontal regeneration and maturation. Therefore, our animal model is recommended to investigate early healing processes that occur after blood coagulation as a consequence of material implantation. Furthermore, it benefits the extraoral approach of surgery by isolating the defect from the oral cavity, which prevents any contamination or infection by saliva and the resident oral flora.^38^

Further investigations using chronic and critical-size defects are recommended to simulate an environment with previous periodontal disease and to confirm the potential influence of BM-MSCs and BMACs in different deliveries on periodontal regeneration.

## Conclusion

MSCs derived from bone marrow promoted bone formation regardless of the form of cell transport, such as BMCA or BM-MSCs. Notably, faster bone formation and maturation and complete periodontal regeneration of fenestration defects, with new PDL fibers inserted into the new cellular cementum tissue, were only possible with the application of cultured BM-MSCs. Our findings, in this preclinical model, highlight important components of the regeneration process, which could help future studies in the direct use of stem cells in periodontal defects or the application of proper products to elicit the host’s periodontium stem cells.

## References

[1] Nuñez J, Vignoletti F, Caffesse RG, Sanz M. Cellular therapy in periodontal regeneration. Periodontol 2000. 2019;79(1):107-16. doi: 10.1111/prd.12250 10.1111/prd.12250 30892768

[2] Tassi SA, Sergio NZ, Misawa MY, Villar CC. Efficacy of stem cells on periodontal regeneration: systematic review of pre-clinical studies. J Periodontal Res. 2017;52(5):793-812. doi: 10.1111/jre.12455 10.1111/jre.12455 28394043

[3] Hynes K, Menicanin D, Gronthos S, Mark Bartold P. Clinical utility of stem cells for periodontal regeneration. Periodontol 2000. 2012;59(1):203-27. doi: 10.1111/j.1600-0757.2012.00443.x 10.1111/j.1600-0757.2012.00443.x 22507067

[4] Dominici M, Le Blanc K, Mueller I, Slaper-Cortenbach I, Marini FC, Krause DS, et al. Minimal criteria for defining multipotent mesenchymal stromal cells. The International Society for Cellular Therapy position statement. Cytotherapy. 2006;8(4):315-7. doi: 10.1080/14653240600855905 10.1080/14653240600855905 16923606

[5] Friedenstein AJ, Piatetzkt -Shapiro II, Petrakova KV. Osteogenesis in transplants of bone marrow cells. J Embryol Exp Morphol. 1966;16(3):381-90. 5336210

[6] Nagata MJH, Santinoni CS, Pola NM, Campos N, Messora MR, Bomfim SR, et al. Bone marrow aspirate combined with low-level laser therapy: a new therapeutic approach to enhance bone healing. J Photochem Photobiol B. 2013;121:6-14. doi: 10.1016/j.jphotobiol.2013.01.013 10.1016/j.jphotobiol.2013.01.013 23474527

[7] Nagata MJ, Campos N, Messora MR, Santinoni CS, Bomfim SR, Fucini SE, et al. Platelet-rich plasma derived from bone marrow aspirate promotes new cementum formation. J Periodontol. 2014;85(12):1702-11. doi: 10.1902/jop.2014.140083 10.1902/jop.2014.140083 25102020

[8] Gessmann J, Köller M, Godry H, Schildhauer TA, Seybold D. Regenerate augmentation with bone marrow concentrate after traumatic bone loss. Orthop Rev (Pavia). 2012;4(1):e14. doi: 10.4081/or.2012.e14 10.4081/or.2012.e14 PMC3348689 22577502

[9] Zhong W, Sumita Y, Ohba S, Kawasaki T, Nagai K, Ma G, et al. *In vivo* comparison of the bone regeneration capability of human bone marrow concentrates vs platelet rich plasma. PLoS One. 2012;7(7):e40833. doi: 10.1371/journal.pone.0040833 10.1371/journal.pone.0040833 PMC3395629 22808272

[10] Li H, Yan F, Lei L, Li Y, Xiao Y. Application of autologous cryopreserved bone marrow mesenchymal stem cells for periodontal regeneration in dogs. Cells Tissues Organs. 2009;190(2):94-101. doi: 10.1159/000166547 10.1159/000166547 18957835

[11] Yang Yi, Rossi FMV, Putnins EE. Periodontal regeneration using engineered bone marrow mesenchymal stromal cells. Biomaterials. 2010;31(33):8574-82. doi: 10.1016/j.biomaterials.2010.06.026 10.1016/j.biomaterials.2010.06.026 20832109

[12] Tsumanuma Y, Iwata T, Washio K, Yoshida T, Yamada A, Takagi R, et al. Comparison of different tissue-derived stem cell sheets for periodontal regeneration in a canine 1-wall defect model. Biomaterials. 2011;32(25):5819-25. doi: 10.1016/j.biomaterials.2011.04.071 10.1016/j.biomaterials.2011.04.071 21605900

[13] Simsek SB, Keles GC, Baris S, Cetinkaya BO. Comparison of mesenchymal stem cells and autogenous cortical bone graft in the treatment of class II furcation defects in dogs. Clin Oral Invest. 2012;16(1):251-8. doi: 10.1007/s00784-010-0486-7 10.1007/s00784-010-0486-7 21086003

[14] Cai X, Yang F, Yan X, Yang W, Yu N, Oortgiesen DAW, et al. Influence of bone marrow-derived mesenchymal stem cells pre-implantation differentiation approach on periodontal regeneration *in vivo*. J Clin Periodontol. 2015;42(4):380–9. doi: 10.1111/jcpe.12379 10.1111/jcpe.12379 25692209

[15] Paknejad M, Eslaminejad MB, Ghaedi B, Rokn A-R, Khorsand A, Etemad-Moghadam S, et al. *Isolat*ion and assessment of mesenchymal stem cells derived from bone marrow: histologic and histomorphometric study in a canine periodontal defect. J Oral Implantol. 2015;41(3):284-91. doi: 10.1563/AAID-JOI-D-13-00220 10.1563/AAID-JOI-D-13-00220 24383495

[16] Nagahara T, Yoshimatsu S, Shiba H, Kawaguchi H, Takeda K, Iwata T, et al. Introduction of a mixture of beta- tricalcium phosphate into a complex of bone marrow mesenchymal stem cells and type I collagen can augment the volume of alveolar bone with- out impairing cementum regeneration. J Periodontol. 2015;86(3):456-64. doi: 10.1902/jop.2014.140384 10.1902/jop.2014.140384 25494830

[17] Novello S, Debouche A, Philippe M, Naudet F, Jeanne S. Clinical application of mesenchymal stem cells in periodontal regeneration: a systematic review and meta-analysis. J Periodont Res. 2020;55(1):1-12. doi: 10.1111/jre.12684 10.1111/jre.12684 31378933

[18] Mu S, Tee BC, Emam H, Zhou Y, Sun Z. Culture-expanded mesenchymal stem cell sheets enhance extraction-site alveolar bone growth: an animal study. J Periodontal Res. 2018;53(4):514-24. doi: 10.1111/jre.12541 10.1111/jre.12541 29633276

[19] Sanz M, Dahlin C, Apatzidou D, Artzi Z, Bozic D, Calciolari E, et al. Biomaterials and regenerative technologies used in bone regeneration in the craniomaxillofacial region: Consensus report of group 2 of the 15th European Workshop on Periodontology on Bone Regeneration. J Clin Periodontol. 2019;46 Suppl 21:82-91. doi: 10.1111/jcpe.13123 10.1111/jcpe.13123 31215114

[20] Jäger M, Herten M, Fochtmann U, Fischer J, Hernigou P, Zilkens C, et al. Bridging the gap: bone marrow aspiration concentrate reduces autologous bone grafting in osseous defects. J Orthop Res. 2011;29(2):173-80. doi: 10.1002/jor.21230 10.1002/jor.21230 20740672

[21] Sauerbier S, Giessenhagen B, Gutwerk W, Rauch P, Xavier SP, Oshima T, et al. Bone marrow aspirate concentrate used with bovine bone mineral to reconstruct vertical and horizontal mandibular defects: report of two techniques. Int J Oral Maxillofac Implants. 2013;28(5):e310-4. doi: 10.11607/jomi.te13 10.11607/jomi.te13 24066349

[22] Santinoni CS, Neves AP, Almeida BF, Kajimoto NC, Pola NM, Caliente EA, et al. Bone marrow coagulated and low-level laser therapy accelerate bone healing by enhancing angiogenesis, cell proliferation, osteoblast differentiation, and mineralization. J Biomed Mater Res A. 2021;109(6):849-58. doi: 10.1002/jbm.a.37076 10.1002/jbm.a.37076 32815657

[23] Melville JC, Tran HQ, Bhatti AK, Manon V, Young S, Wong ME. Is reconstruction of large mandibular defects using bioengineering materials effective? J Oral Maxillofac Surg. 2020;78(4):661.e1-661.e29. doi: 10.1016/j.joms.2019.11.024 10.1016/j.joms.2019.11.024 31883442

[24] Hernigou PH, Poignard A, Beaujean F, Rouard H. Percutaneous autologous bone-marrow grafting for nonunions. Influence of the number and concentration of progenitor cells. J Bone Joint Surg Am. 2005;87(7):1430-7. doi: 10.2106/JBJS.D.02215 10.2106/JBJS.D.02215 15995108

[25] Kilkenny C, Browne WJ, Cuthill IC, Emerson M, Altman DG. Improving bioscience research reporting: the ARRIVE guidelines for reporting animal research. PLoS Biol. 2010;8(6):e1000412. doi: 10.1371/journal.pbio.1000412 10.1371/journal.pbio.1000412 PMC2893951 20613859

[26] Gregory CA, Gunn WG, Peister A, Prockop DJ. An Alizarin red-based assay of mineralization by adherent cells in culture: comparison with cetylpyridinium chloride extraction. Anal Biochem. 2004;329:77-84. doi: 10.1016/j.ab.2004.02.002 10.1016/j.ab.2004.02.002 15136169

[27] Livak KJ, Schmittgen TD. Analysis of relative gene expression data using real-time quantitative PCR and the 2(−Delta C(T)) method. Methods. 2001;25(4):402-8. doi: 10.1006/meth.2001.1262 10.1006/meth.2001.1262 11846609

[28] Abuna RP, Oliveira FS, Santos TD, Guerra TR, Rosa AL, Beloti MM. Participation of TNF-α in inhibitory effects of adipocytes on osteoblast differentiation. J Cell Physiol. 2016;231(1):2014-14. doi: 10.1002/jcp.25073 10.1002/jcp.25073 26059069

[29] King GN, King N, Cruchley AT, Wozney JM, Hughes FJ. Recombinant human bone morphogenetic protein-2 promotes wound healing in rat periodontal fenestration defects. J Dent Res. 1997;76(8):1460-70. doi: 10.1177/00220345970760080801 10.1177/00220345970760080801 9240382

[30] Oliveira PG, Souza AM, Novaes AB Jr, Taba M Jr, Messora MR, Palioto DB, et al. Adjunctive effect of antimicrobial photodynamic therapy in induced periodontal disease. Animal study with histomorphometrical, immunohistochemical, and cytokine evaluation. Lasers Med Sci. 2016 Sep;31(7):1275-83. doi: 10.1007/s10103-016-1960-5 10.1007/s10103-016-1960-5 27351664

[31] Benatti BB, Neto JBC, Casati MZ, Sallum EA, Sallum AW, Nociti FH Jr . Periodontal healing may be affected by aging: a histologic study in rats. J Periodontal Res. 2006;41(4):329-33. doi: 10.1111/j.1600-0765.2006.00872.x 10.1111/j.1600-0765.2006.00872.x 16827728

[32] Correa MG, Campos ML, Marques MR, Casati MZ, Nociti Jr FH, Sallum EA. Histometric analysis of the effect of enamel matrix derivative on the healing of periodontal defects in rats with diabetes. J Periodontol. 2013;84(9):1309-18. doi: 10.1902/jop.2012.120354 10.1902/jop.2012.120354 23121457

[33] Correa MG, Campos ML, Marques MR, Ambrosano GM, Casati MZ, Nociti FH Jr, et al. Outcome of enamel matrix derivative treatment in the presence of chronic stress: histometric study in rats. J Periodontol. 2014;85(7):e259-e267. doi: 10.1902/jop.2013.130383 10.1902/jop.2013.130383 24283657

[34] Rodrigues TL, Nagatomo KJ, Foster BL, Nociti FH, Somerman MJ. Modulation of phosphate/pyrophosphate metabolism to regenerate the periodontium: a novel *in vivo* Approach. J Periodontol. 2011;82(12):1757-66. doi: 10.1902/jop.2011.110103 10.1902/jop.2011.110103 PMC3884815 21488756

[35] Nishimoto S, Oyama T, Matsuda K. Simultaneous concentration of platelets and marrow cells: a simple and useful technique to obtain source cells and growth factors for regenerative medicine. Wound Repair Regen. 2007;15(1):156-62. doi: 10.1111/j.1524-475X.2006.00196.x 10.1111/j.1524-475X.2006.00196.x 17244331

[36] Jäger A, Götz W, Lossdörfer S, Rath-Deschner B. Localization of SOST/sclerostin in cementocytes *in vivo* and in mineralizing periodontal ligament cells *in vitro*. J Periodont Res. 2010;45(2):246-54. doi: 10.1111/j.1600-0765.2009.01227.x 10.1111/j.1600-0765.2009.01227.x 19778325

[37] Kawaguchi H, Hirachi A, Hasegawa N, Iwata T, Hamaguchi H, Shiba H, et al. Enhancement of periodontal tissue regeneration by transplantation of bone marrow mesenchymal stem cells. J Periodontol. 2004;75(9):1281-7. doi: 10.1902/jop.2004.75.9.1281 10.1902/jop.2004.75.9.1281 15515346

[38] Rios F, Giannobile W. Preclinical protocols for periodontal regeneration. In: Giannobile WV, Nevins M, editors. Osteology guidelines for oral and maxillofacial regeneration: preclinical models for translational research. London: Quintessence; 2011. p. 77-102.

